# The Stimulative Effect of Yangjing Capsule on Testosterone Synthesis through Nur77 Pathway in Leydig Cells

**DOI:** 10.1155/2015/408686

**Published:** 2015-08-27

**Authors:** Yalong Gu, Xindong Zhang, Dalin Sun, Hongle Zhao, Bin Cai, Chao Gao, Li Gao, Yugui Cui, Zhian Tang, Baofang Jin

**Affiliations:** ^1^Andrology Department of Integrative Medicine, Zhongda Hospital, School of Medicine, Southeast University, Nanjing 210009, China; ^2^Reproductive Medicine Center, Department of Obstetrics and Gynecology, Nanjing Drum Tower Hospital, Nanjing University Medical School, Nanjing 210008, China; ^3^State Key Laboratory of Reproductive Medicine, Clinical Center of Reproductive Medicine, First Affiliated Hospital, Nanjing Medical University, Nanjing 210029, China; ^4^Department of Traditional Chinese Medicine, Yixing People's Hospital, Yixing, Jiangsu 214200, China

## Abstract

Yangjing Capsule (YC), an innovative Chinese medicine based on traditional prescription, promotes testosterone synthesis by upregulating the expression of steroidogenic enzymes. Nur77 as a nuclear receptor is known to regulate the expression of many steroid synthetases. This study aimed to explore the potential mechanisms by which YC regulates testosterone synthesis in Leydig cells. Real-time PCR and Western blot analysis were employed to assess the expressions of steroidogenic enzymes and Nur77 after treating MLTC-1 cells with YC. The luciferase reporter gene assay was performed to detect the activity of Nur77 gene promoter. Also, the expressions of steroid synthases were detected after Nur77 gene was knocked down. YC significantly stimulated Nur77 production and upregulated StAR and HSD3B expression, and this agrees with the activity of Nur77 gene promoter that was significantly enhanced by YC. Interestingly, knockdown of Nur77 blocked the above YC's effects and consequently inhibited testosterone synthesis in MLTC-1 cells. YC promotes StAR and HSD3B expression and upregulates testosterone synthesis in Leydig cells, which is mediated by Nur77 pathway.

## 1. Introduction

Testosterone, the predominant circulating androgen, is critical for the regulation of various vital processes such as male sexual differentiation, maintenance of spermatogenesis, and expression of male secondary sex characteristics [1]. Testicular Leydig cells are the main androgen-producing cells in mammals. Testosterone synthesis in Leydig cells is a multistep process that requires the well-orchestrated action of several hormones and signaling molecules produced by different endocrine and paracrine/autocrine cells [2]. In response to these signals, several transcription factors are activated, which results in upregulating the expressions of many genes involved in steroidogenesis, eventually leading to testosterone synthesis by Leydig cells.

The orphan nuclear receptor Nur77 (also termed NGFI-B, TR3, or NR4A1) is a transcription factor that belongs to the nuclear hormone receptor superfamily [3–5]. Nur77 is an early response gene identified originally through its rapid activation by nerve growth factor (NGF) in PC12 pheochromocytoma cells and fibroblasts [6]. Indeed, Nur77 expression is rapidly and strongly induced by various stimuli in numerous tissues, including hormonally stimulated steroidogenic cells [7–10]. The monomeric or dimeric Nur77 is an endpoint effector of the protein kinase A (PKA) signaling pathway. The activation function-1 domain of Nur77 plays a major role in transcriptional activation, cofactor recruitment, and intra- and intermolecular interactions [11, 12]. Regarding its role in transcriptional activation, Nur77 has been shown to regulate genes involved in steroidogenesis, including StAR and HSD3B in Leydig cells [7, 13].

YC is composed of Herba Epimedii Brevicornus, Radix Rehmanniae Preparata, Rhizoma Polygonati Sibirici, Placenta Hominis, Angelica Sinensis, and other components. It has been shown that YC promotes testosterone synthesis via steroidogenic enzymes (CYP11A1 and HSD3B) and StAR and can be used for the treatment of male infertility and sexual dysfunction [14, 15]. However, the molecular mechanisms by which YC affects the expression of steroidogenic enzymes and promotes testosterone synthesis remain unclear.

In the present study, we demonstrated that YC promotes testicular steroidogenesis, at least in part, by upregulating the orphan nuclear receptor Nur77. These findings provide a molecular mechanism for YC-mediated induction of testosterone production in testicular Leydig cells.

## 2. Materials and Methods

### 2.1. Chemicals

RPMI 1640 medium, fetal bovine serum (FBS), and lyophilized trypsin-EDTA were obtained from GIBCO BRL (Grand Island, NY, USA). Dimethyl sulfoxide (DMSO), human chorionic gonadotropin (hCG), diethylpyrocarbonate (DEPC), sodium dodecyl sulfate (SDS), and Tris/HCl were purchased from Sigma (St. Louis, MO, USA). Whole protein extraction kit was purchased from Keygen Biotech Co. Ltd. (Nanjing, China). PrimeScript RT Master Mix and SYBR Green PCR Master Mix reagent kits were obtained from TaKaRa (TaKaRa Biotechnology, Dalian, China). The primers used in this study were synthesized by Invitrogen Life Tech (Carlsbad, CA, USA). The BCA Protein Assay kit was purchased from Beyotime (Beyotime Institute of Biotechnology, Shanghai, China). PageRuler Prestained Protein Ladder was obtained from Thermo Fisher Scientific (Thermo Fisher Scientific, Shanghai, China). Rabbit polyclonal anti-Nur77, rabbit polyclonal anti-HSD3B, mouse polyclonal anti-StAR, and mouse polyclonal anti-GAPDH antibodies were purchased from Abcam (Cambridge Science Park, Cambridge, UK). HRP-conjugated secondary antibodies were from Jackson ImmunoResearch (Baltimore, PA, USA). Enhanced chemiluminescence kit was obtained from Amersham Biosciences (Uppsala, Sweden).

### 2.2. Preparation of YC and Cells

YC was prepared as previously described [15]. YC content (3.33 g, equivalent to 10 g of crude drug) was extracted with 333 mL of distilled water and subsequently subjected to ultrasonic extraction for 45 min. The supernatant was collected, and the precipitate was dissolved and extracted in a similar manner. The two solutions were combined and centrifuged at 13,000 g and 4°C for 30 min. The resulting supernatant was concentrated to 100 mL with a rotary evaporator at 60°C. The final YC corresponded to 100 mg/mL of the crude herbal dose. No hCG or estradiol was detected in the extract by radioimmunoassay (RIA). After pH adjustment to 7.0, the extract was sterilized by filtration and stored at −70°C until use. MLTC-1 cells constitute a useful cellular model for studying steroidogenesis and regulation. They were obtained from the Cell Institute of Shanghai (Shanghai, China). MLTC-1 cells were cultured in RPMI-1640 containing 10% heat-inactivated fetal bovine serum in 5% CO_2_ at 37°C. Pre-confluent (70–75%) MLTC-1 cells in 6-well plates were treated with 1 mg/mL YC for 0, 1, 2, 4, and 8 h, respectively. In addition, cells were treated with 0, 0.01, 0.1, and 1 mg/mL YC and 0.1 U/mL hCG (used as positive control) for 2 h. After treatment, cells were harvested. Nur77 mRNA and protein expression levels in cells were determined by real-time PCR and Western blot.

### 2.3. Transient Transfection and Luciferase Reporter Gene Assays

The mouse −1604 bp Nur77 promoter sequence was amplified by PCR from mouse genomic DNA with the primers 5′-GCTACTCGAGCTCTGAACATATCACCGAAT-3′ and 5′-TAGCAATCTTCTCCGCAGTCCTTCTAGCACA-3′. Deletion of the Nur77 promoter to −710 bp was obtained by PCR using the −1604 bp Nur77 promoter as template, along with a common reverse primer: 5′-TAGCAATCTTCTCCGCAGTCCTTCTAGCACA-3′ and forward primer: 5′-TATAGAGGGGAAGGAACCTTGAAGGCCA-3′. The PCR products were cloned into the pGL3-basic luciferase reporter plasmid (Promega, Madison, WI, USA) and sequenced for confirmation. Pre-confluent (70–75%) MLTC-1 cells in 24-well plates were transfected with 200 ng Nur77 promoter-Luc or the internal control promoter (10 ng phRL-TK Renilla luciferase expression vector). Twenty-four hours after transfection, cells were treated with 0, 0.01, 0.1, and 1 mg/mL YC and 0.1 U/mL hCG (used as positive control). Luciferase activity was assessed 24 h after YC treatment using the Dual Luciferase Assay System (Promega, Madison, WI, USA).

### 2.4. Adenovirus Construction and Infection

Adenoviruses containing Nur77-targeted siRNA (Ad-siNur77) and a control Ad-CMV-GFP construct (Ad-CMV) were generated by Shanghai Ji Kai gene Chemical Technology Co., Ltd., respectively, as described previously [16]. Twenty-four hours before transfection, MLTC-1 cells were plated in 6-well culture dishes at a density of 2 × 10^5^ cells per well. Transfection was performed using Ad-siNur77 or Ad-CMV at an MOI of 100. At 48 h after transfection, cells were treated with or without 1 mg/mL YC for 4 h and collected for RNA and protein purification for real-time PCR and Western blot experiments.

### 2.5. Hormone Assessment Assays

For YC treatment, exponentially growing MLTC-1 cells were seeded in 6-well plates at 2 × 10^5^/well and cultured for 24 h. Transfection was performed with recombinant adenovirus Ad-siNur77 (MOI 100) or Ad-CMV (MOI 100). After 48 h transfection, cells were treated with or without YC (1 mg/mL) for 24 h, and the cell culture medium was collected for RIA.

### 2.6. RNA Isolation and Real-Time PCR

Total RNA was extracted using Trizol reagent. cDNA was synthesized from 1 mg total RNA using a PrimeScript RT Master Mix. The following specific primers were used for PCR: Nur77, forward: 5′-GAC CCC ACT ATT TGT CTT ATC CC-3′ and reverse: 5′-CCC ATC TCA ACC TCT TGC TTT C-3′. StAR, forward: 5′-CGG GTG GAT GGG TCA AGT TC-3′ and reverse: 5′-GCA CTT CGT CCC CGT TCT C-3′. HSD3B, forward: 5′-GTG GGG CTT CTG CCT TGA T-3′ and reverse: 5′-GGT TTT CTG CTT GGC TTC CTC-3′. GAPDH, forward: 5′-AGG TTG TCTCCT GCG ACT TCA-3′ and reverse: 5′-GGG TGG TCC AGG GTT TCT TAC T-3′. The reactions were performed at 95°C for 5 min followed by 40 cycles at 95°C for 30 s and 60°C for 60 s. Melting curve analysis was performed to confirm the products. The relative abundance of the target mRNAs was calculated using the 2^−ΔΔCt^ method. The data were expressed as a percentage of control set to 100%.

### 2.7. Protein Extraction and Western Blot Analysis

Cells were harvested, washed three times with precooled PBS, and treated with cell lysis buffer. Protein samples were prepared and separated using sodium dodecyl sulfate polyacrylamide gel electrophoresis (SDS-PAGE) as previously described [15]. Briefly, equal amounts of total protein (40 *μ*g) were separated by SDS-PAGE using 10% gel and then transferred onto nitrocellulose membranes. Immunoblotting was performed with primary antibodies against Nur77 (1 : 1000), StAR (1 : 1000), and HSD3B (1 : 1000). GAPDH (1 : 5000) was used as an internal control. Immunodetection was carried out using goat anti-rabbit (1 : 5000) or goat anti-mouse (1 : 5000) secondary antibodies and enhanced chemiluminescence detection kit.

### 2.8. Statistical Analysis

Data are presented as mean ± SD of values from at least three independent experiments. Differences among groups were assessed by one-way ANOVA followed by least significance difference (LSD) or *t*-test. *P* < 0.05 was considered statistically significant.

## 3. Results

### 3.1. Effects of YC on Nur77 Expression

**Figure 1:**
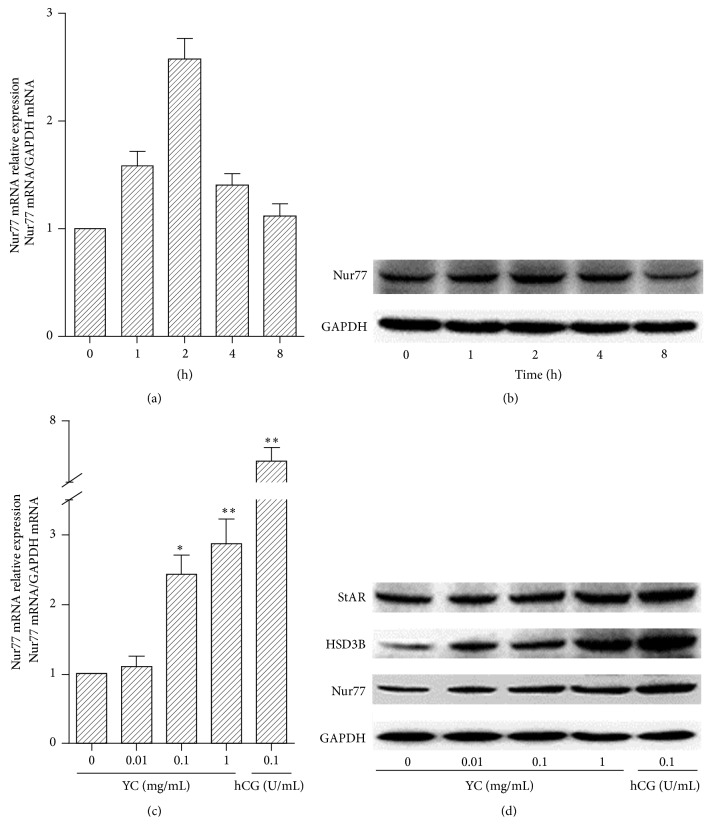
Effects of YC on Nur77 mRNA and protein expression in MLTC-1 cells. (a and b) MLTC-1 cells were treated with YC (1 mg/mL) for 0 h, 1 h, 2 h, 4 h, and 8 h, respectively. (c and d) MLTC-1 cells were treated with 0, 0.01, 0.1, and 1 mg/mL YC and 0.1 U/mL hCG for 2 h. mRNA and protein expression levels were assessed by real-time PCR and Western blot, respectively. Data are a percentage of the control set to 100%. The values are mean ± SD from three independent experiments. Compared with the control, ^*∗*^ *P* < 0.05 and ^*∗∗*^ *P* < 0.01.

As shown in Figures 1(a) and 1(b), YC (1 mg/mL) treatment caused a transient increase in Nur77 mRNA expression, which reached a maximum level, 2.6-fold the basal amounts, within 2 h before returning to the basal level after 8 h. Nur77 protein induction was dramatically increased within 2 h of YC treatment and peaked at 4 h after YC treatment. When MLTC-1 cells were treated with YC at different concentrations (0~1 mg/mL), Nur77 expression was rapidly induced both at the mRNA and protein levels in a dose-dependent manner (Figures 1(c) and 1(d)). Also, StAR and HSD3B protein expressions were induced by YC with a dose-course pattern that was similar to that of Nur77 expression (Figure 1(d)). These results suggested that YC could affect cell function through Nur77.

### 3.2. Effects of YC on Nur77 Promoter Activity

**Figure 2:**
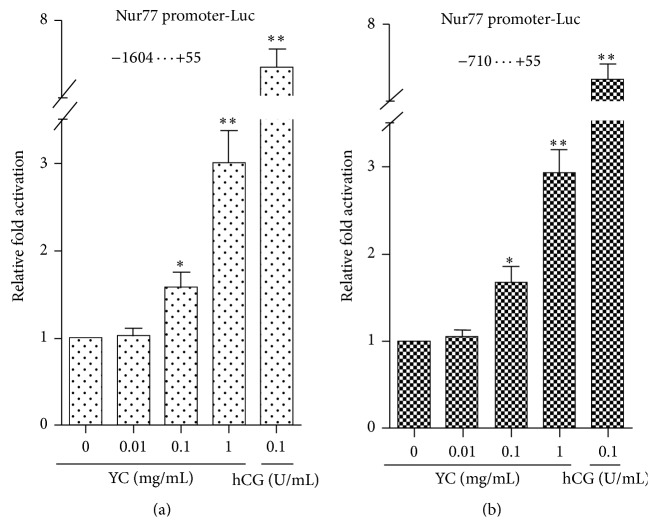
Effects of YC on Nur77 promoter activity in MLTC-1 cells. MLTC-1 cells were transfected with 200 ng Nur77 promoter-Luc. Cells were treated with 0, 0.01, 0.1, and 1 mg/mL YC and 0.1 U/mL hCG and assayed for luciferase activity after 24 h. Luciferase activity was normalized by 10 ng phRL-TK Renilla luciferase expression vector to determine the transfection efficiency. Data are a percentage of the control set to 100%. Values are mean ± SD from three independent experiments. Compared with the control, ^*∗*^ *P* < 0.05 and ^*∗∗*^ *P* < 0.01.

To test whether YC could directly regulate Nur77 promoter activity in steroidogenic cells, we performed luciferase reporter gene assays in MLTC-1 cells (Figure 2). Nur77 gene promoter was induced by YC treatment in a dose-dependent manner, and its activity increased by 1.6- to 3-fold compared with the control group. The response of the Nur77 promoter (bp −1604 to bp +55 or/and bp −710 to bp +55) to YC in transient transfection was consistent with the mRNA expression pattern observed in MLTC-1 cells. These results indicated that transcriptional regulation of Nur77 was associated with the YC-mediated Nur77 expression.

### 3.3. Nur77 Knockdown Blocks YC-Induced Steroidogenesis in MLTC-1 Cells

**Figure 3:**
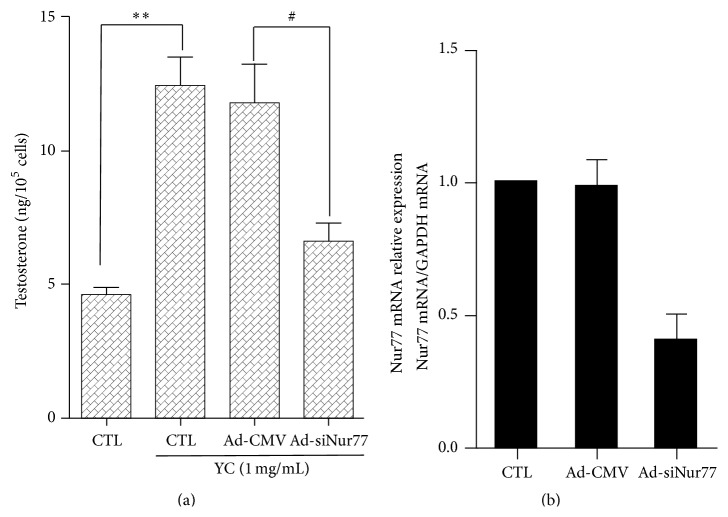
Nur77 knockdown blocks YC-induced testosterone synthesis in MLTC-1 cells. MLTC-1 cells were exposed to control blank, 1 mg/mL YC and 1 mg/mL YC with or without Ad-siNur77 (MOI 100). Then, the culture medium was collected for testosterone concentration measurements by RIA. Values are mean ± SD from three independent experiments. ^*∗∗*^ *P* < 0.01 compared with the control group; ^#^ *P* < 0.05 compared with YC and Ad-CMV groups.

To test the possibility of Ad-siNur77 repressing Nur77 transcription, Nur77 expression patterns were compared after treating MLTC-1 cells with Ad-siNur77. As shown in Figure 3(b), Ad-siNur77 maintained an interference efficiency of about 50%. Then after the treatment, the culture media collected from MLTC-1 cells were transiently expressing Ad-siNur77. These culture media were used to quantify testosterone amounts by RIA (Figure 3(a)). Testosterone synthesis was significantly induced by approximately 2.5-fold in YC-treated (1 mg/mL) MLTC-1 cells after 24 h when compared with the control group. Knockdown of Nur77 decreased the YC-induced testosterone synthesis by approximately 40%. These results suggested that Nur77 could be involved in the YC-mediated enhancement of testosterone synthesis.

### 3.4. Nur77 Knockdown Blocks YC-Induced Upregulation of StAR and HSD3B

**Figure 4:**
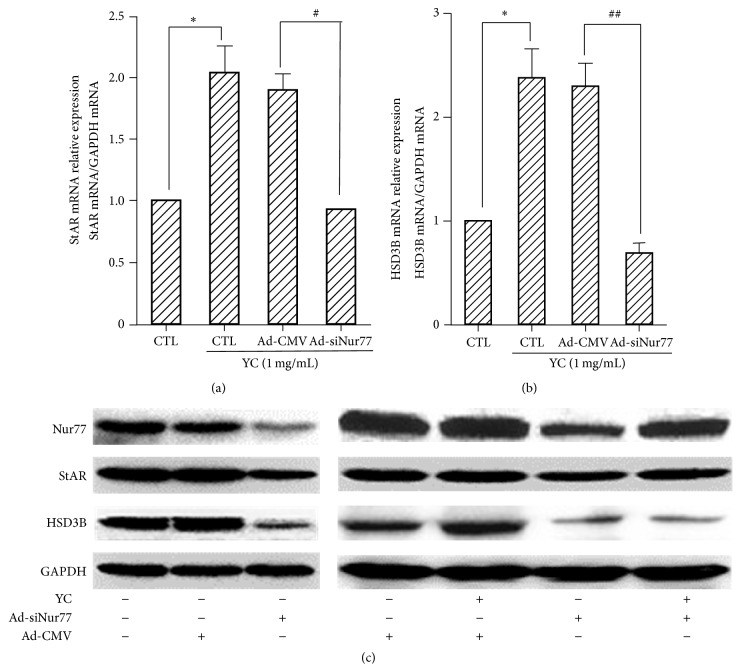
Nur77 knockdown reduces the YC-mediated induction of StAR and HSD3B. MLTC-1 cells were exposed to control blank, 1 mg/mL YC and 1 mg/mL YC with or without Ad-siNur77 at an MOI of 100. Gene and protein expression levels were detected by real-time PCR and Western blot, respectively. Data are a percentage of the control set to 100%. Values are mean ± SD from three independent experiments. ^*∗*^ *P* < 0.05 compared with the control group; ^##^ *P* < 0.05, ^##^ *P* < 0.01 compared with YC and Ad-CMV groups.

As shown in Figure 4, Ad-siNur77 transfection resulted in a 51% decrease in StAR mRNA levels (Figure 4(a)) and 72% reduction in HSD3B mRNA expression (Figure 4(b)) after 2 h of treatment with 1 mg/mL YC. Meanwhile, StAR and HSD3B protein levels in response to YC stimulation were also decreased in MTC-1 cells after transfection with Ad-siNur77. These data indicated that Nur77 should contribute to the induction of StAR and HSD3B expression in response to YC in Leydig cells.

## 4. Discussion

Yangjing Capsule enhances libido and erections in patients with male sexual disorders, possibly by promoting testosterone synthesis [14, 15]. Although the physiological roles of YC in the mammalian reproductive function are well established, very little is known about the molecular mechanisms by which it regulates the downstream pathways on gonadal cells. Here we reported a transient and rapid induction of Nur77 expression, following the alteration of steroidogenesis, in the YC-treated MLTC-1 cells.

Nur77 has recently received the increasing attention as an important regulator of basal and hormone-induced gene expression in steroidogenic cells, including testicular Leydig cells. Indeed, Nur77 expression is rapidly induced in testicular Leydig cells in response to LH/FSK/cAMP [7, 8, 13], which are well-known regulators of Leydig cell gene expression and function. Our results showed that the Nur77 expression in MLTC-1 cells was also induced by YC, in dose- and time-dependent manners. A report on LH-mediated Nur77 induction described similar findings [8]. Therefore, YC and LH might share a similar function in cells.

LH/hCG controls Leydig cell function under physiological condition via its specific receptor (LHR) that is coupled to both adenylate cyclase and phospholipase C pathways [17]. Exposure of Leydig cells to LH/hCG causes a long-term trophic effect of the hormone, which involves increased transcription and translation of genes encoding essential components of steroidogenesis. In long-term response, the transcription factor Nur77 promoter is activated by LH/hCG-mediated cAMP-PKA signaling pathway, which is necessary for maximal steroidogenesis [18, 19]. Based on the comparable effects of YC and LH/hCG, it is reasonable to speculate that YC regulates Nur77 expression at the transcriptional level. To test this hypothesis, transient transfection experiments with Nur77 promoter fragment (bp −1604 to bp +55 and bp −710 to bp +55) were carried out, and we found that YC induced the activity of Nur77 promoter. Multiple cis-acting elements in this promoter region, such as CREB, AP-1 like elements, NF-*κ*B-p50, and C/EBP*β*, have been previously characterized [20, 21]. Therefore, multiple transcription factors may be involved in the YC-mediated Nur77 induction in Leydig cells.

The orphan nuclear receptor Nur77 participates in the hormonal stimulation of gene expression in testicular Leydig cells [7, 22–25]. Indeed, Nur77 has been shown to activate the promoters of some steroidogenic genes (Cyp17, Cyp11B2, Cyp21, HSD3B, and StAR) [3, 7, 13, 26, 27], suggesting that Nur77 might have participated in the regulation of steroidogenesis in Leydig cells. Considering that YC upregulated the Nur77 expression, we hypothesized that Nur77 could play a key role in the YC-mediated steroidogenesis. To test this hypothesis, we used Ad-siNur77 to knock down Nur77 expression in MLTC-1 cells. Our results showed that knockdown of Nur77 suppressed the YC-induced testosterone synthesis, suggesting that YC exerted biological effects partly via Nur77. YC, an innovative Chinese medicine based on traditional prescription, consists of 11 Chinese herbals. Because of the complexity of its composition, it was not clear whether the Nur77 selection was achieved by a single or multiple components of YC. Our results demonstrate that YC regulated androgen synthesis through Nur77 pathway and thus provided a theoretical basis for the clinical application of YC. The expression of steroidogenic enzymes is a crucial step in steroid synthesis. The current study demonstrated that StAR and HSD3B expressions were upregulated by YC. Since Nur77 expression was increased in the YC-treated MLTC-1 cells, it is reasonable to speculate that Nur77 could be implicated in the induction of StAR and HSD3B gene expression. Interestingly, the upregulated StAR and HSD3B expressions in the Ad-siNur77 transfected cells were downregulated by YC treatment (Figure 4).

## 5. Conclusions

In summary, Nur77 expression is upregulated by YC in the testicular Leydig cell line in time- and dose-dependent manners and this could mediate the effects of YC in androgen production in testicular Leydig cells. The identification of Nur77 as a target of YC might help us further understand the complicated mechanisms of YC in the treatment of male reproductive diseases.
